# Influence of Hybrid Sol-Gel Crosslinker on Self-Healing Properties for Multifunctional Coatings

**DOI:** 10.3390/ma14185382

**Published:** 2021-09-17

**Authors:** Guillaume Lollivier, Marie Gressier, Florence Ansart, Maëlenn Aufray, Marie-Joëlle Menu

**Affiliations:** 1 CIRIMAT, Université de Toulouse, UPS, 31062 Toulouse, France; lollivier@chimie.ups-tlse.fr (G.L.); gressier@chimie.ups-tlse.fr (M.G.); ansart@chimie.ups-tlse.fr (F.A.); 2 CIRIMAT, Université de Toulouse, INP-CNRS, ENSIACET, 31062 Toulouse, France; maelenn.aufray@ensiacet.fr

**Keywords:** multifunctional material, self-healing coating, anticorrosion coating, hybrid material, nanocomposite, disulfide bond, organosilane

## Abstract

Self-healing polymers are a new class of material that has recently received a lot of attention because of the lifespan improvement it could bring to multiple applications. One of the major challenges is to obtain multifunctional materials which can self-heal and exhibit other interesting properties such as protection against corrosion. In this paper, the effect of the incorporation of an aminosilane on the properties of a self-healing organic polymer containing disulfide bond is studied on films and coatings for aluminium AA2024-T3 using simple one step in situ synthesis. Hybrid coatings with enhanced anticorrosion properties measured by EIS were obtained thanks to the formation of a protective oxide interface layer, while exhibiting wound closure after exposition at 75 °C. The thermal, mechanical and rheological properties of the films with different aminosilane amounts were characterized in order to understand the influence of the slight presence of the inorganic network. Stiffer and reprocessable hybrid films were obtained, capable to recover their mechanical properties after healing. The nanocomposite structure, confirmed by TEM, had a positive effect on the self-healing and stress relaxation properties. These results highlight the potential of sol-gel chemistry to obtain efficient anticorrosion and self-healing coatings.

## 1. Introduction

**Figure 1:**
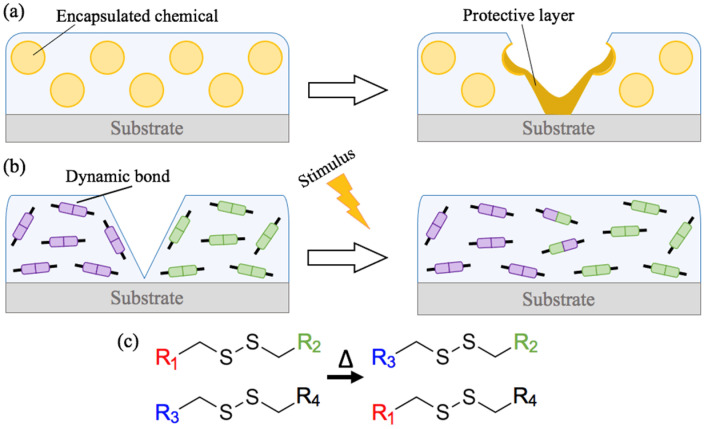
Illustration of (**a**) extrinsic and (**b**) intrinsic strategies to obtain self-healing coatings and (**c**) disulfide metathesis reaction.

Self-healing polymers are a recent class of materials which are capable of recovering their initial properties after damage and are highly desirable in order to obtain reprocessable materials with extended lifetime. Many different strategies to obtain such interesting properties are reported in the literature [1,2,3]. They can be divided into two main categories, denoted extrinsic and intrinsic self-healing materials, schematically represented in Figure 1. Extrinsic self-healing is based on the incorporation of encapsulated chemical compounds in a matrix, corrosion inhibitors or polymerisable materials for example, that will be released upon damage and will polymerize to ensure sufficient cohesive strength to the structure (Figure 1a) [4,5]. Intrinsic self-healing relies on the capacity of a material to repair itself without any external agents, autonomously or by activation using an external trigger (Figure 1b). This strategy is based on the presence of so-called “dynamic bonds” in the polymeric network. Those bonds, covalent or not, are able to recombine to reconstruct the wounded polymeric network [6,7].

A wide variety of studies on intrinsic self-healing, also called adaptive covalent network polymers has been published since their discovery by Leibler in 2011 [8], based on different recombination reactions using disulfide metathesis (Figure 1c) [9], hydrogen bonds [10,11] or the Diels–Alder reversible reaction, for example [12]. Lafont [13], Pepels [14], Zadeh [15,16] and more recently Song [17] described self-healing polymers based on disulfide-thiol exchange reactions, easy to synthetize and capable of recovering their mechanical properties after exposition to heat. The main objective of the researches on the subject at the moment is to obtain performant multifunctional materials, being able not only to heal but also with other properties such as electrical conductivity or mechanical resistance, in order to allow their possible use in industrial applications. Diverse properties have already been obtained for such materials, useful in a wide range of applications from strain sensors [18] to protective coatings [15,19]. Performant self-healing anticorrosive coatings could be an interesting alternative to previous toxic chromated surface treatments thanks to their extended lifetime protection offered by the recovery of their barrier properties after damage.

Because of the regulations and restrictions on surface treatments based on chromium (VI) in the European Union [20], performant and eco-friendly anticorrosion protective coatings are needed. A lot of research based on organic or inorganic materials has already been published on the subject in the last 20 years. Some of the most promising results are obtained with organic–inorganic hybrid materials, combining the benefits brought by each chemical part [21,22]. Those materials are often synthesized using hybrid alkoxysilanes precursors such as 3-aminopropyltriethoxysilane (APTES) or (3-glycidyloxypropyl)trimethoxysilane (GPTMS), sometimes called silane coupling agents [23]. Those molecules are composed of an alcoxysilane moiety, -Si(OR)_3_ (R = Me, Et), able to condense through sol-gel reactions to create a siloxane inorganic network, and an organic function linked to the same silicon atom which can interact with or create an organic polymeric network. Their ability to connect both phases permits to obtain strong interconnected hybrid materials, combining the resistance of the inorganic coatings with the flexibility of the organic ones [22]. Their use in nanocomposite materials can also improve the thermal and mechanical properties thanks to the better miscibility between phases [24]. Bakhshandeh et al. [25] highlighted for example the interest of involving organic-inorganic precursor such as APTES to obtain strong anticorrosion hybrid coatings based on DGEBA derived epoxy-silica nanocomposite.

Inspired by these studies, this work aims to obtain eco-friendly coatings exhibiting at the same time self-healing and anticorrosion properties on AA2024-T3 aluminium alloy. Polymerization reactions were first monitored using IR, NMR and Raman spectroscopies and the effect of the incorporation of 3-aminopropyltriethoxysilane (APTES) on the properties of a disulfide-based self-healing epoxy polymer was observed using suitable characterization methods. The self-healing properties of the coatings were observed through wounds healing and their anticorrosive properties were assessed using electrochemical impedance spectroscopy (EIS) measurements. The self-healing properties of the films were evaluated using tensile tests and stress relaxation experiments, while the effect of the inorganic phase on the thermal properties was analyzed by TGA and DSC experiments. Those characterizations enabled a complete estimation of the effect of the nanocomposite structure of the hybrid materials, assessed by TEM observations.

## 2. Materials and Methods

### 2.1. Materials and Synthesis

**Figure 2:**
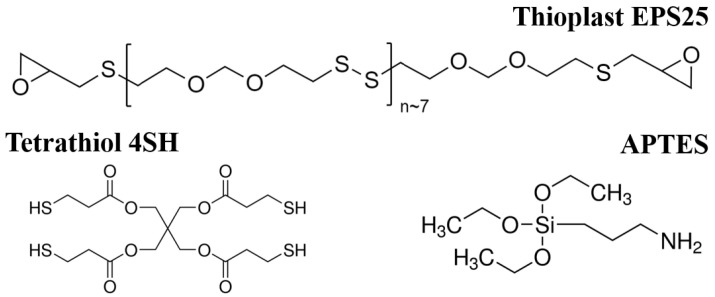
Semi-developed formulas of the epoxy monomer (Thioplast EPS25) and the crosslinkers (4SH and APTES).

Aluminium AA2024-T3 80 × 42 × 1 mm^3^ sheets were used as substrate. Thioplast EPS25 samples (Thioplast, EEW 900 g/eq) was furnished by AkzoNobel (Amsterdam, Netherlands). Epoxy equivalent weight (EEW) was determined using size exclusion chromatography (SEC). Pentaerythritoltetrakis(3-mercaptopropionate) (4SH), 4-(dimethylamino)pyridine (DMAP), (3-aminopropyl)triethoxysilane (APTES) and ethylacetate (EtOAc) were all purchased from Sigma-Aldrich (St. Louis, MO, USA). Chemical formulas are detailed in Figure 2. All chemicals were used without further purification.

Different formulations, resumed in Table 1, were prepared by mixing adequate amounts of Thioplast, 4SH and APTES in EtOAc. 0.1 w% of DMAP was used as a catalyst for thiol-epoxy crosslinking reactions. Relative amounts of APTES compared to 4SH were adjusted in order to evaluate the influence of the hybrid sol-gel crosslinker. Molar ratio of APTES over total crosslinker quantity (APTES + 4SH) denoted H is used for this purpose. It can be associated with the inorganic ratio present in the material and will be used as the denominator for each material used in this study. Four different formulations are used: H0 represents the organic material used for comparison and H30 and H50 being the hybrid materials with an increasing inorganic content. Formulations were designed assuming that only the primary amine function of APTES reacts with epoxide functions of the Thioplast. As the secondary amine functions can also react with epoxides in the same but less favorable way mainly due to steric hindering, H50-2Epox film was synthesized in stoichiometric conditions assuming that both primary and secondary amines will react to confirm this assumption.

Solutions were mixed using a magnetic stirrer for 10 min and then coatings were performed by dipping aluminium AA2024-T3 sheets into the solution using an automated NIMA dip coater (Biolin Scientific, Västra Frölunda). Substrates were degreased in alkaline solution and etched with SOCOSURF A1858 and A1806 solutions prior deposition. Two consecutive dips at 300 mm/min were realized to ensure an average thickness of approximately 30 µm. After deposition, samples were dried and cured at 75 °C during 5 h in an oven.

To investigate the intrinsic changes in the materials properties, xerogels films were obtained by pouring 20 mL of the solution left after dip-coating process in flat Teflon-covered molds. The same thermal treatment as the coatings was used.

### 2.2. Characterization

^13^C and ^29^Si magic angle spinning nuclear magnetic resonance (MAS-NMR) spectra were recorded using a Bruker Avance III 400WB (9.4 T) spectrometer (Toulouse, France). Chemical shift references are tetramethylsilane (TMS) and a 4 mm zirconia rotor was used. Rotation speed around magic angle (MAS) was 8 kHz and experiments were realized at room temperature. ^13^C{^1^H} nuclear magnetic resonance spectra were recorded using a Bruker Avance III 500 MHz spectrometer (Toulouse, France). D6 deuterated acetone was used as internal chemical shift reference. Raman spectroscopy was undertaken using a LabRAM HR 800 Raman confocal spectrometer (Horiba, Kyoto, Japan) with a confocal hole of 10 µm, a 600 lines/mm holographic grating, an opacity filter and a helium-neon laser emitting at 633 nm. Near-infrared spectroscopy was undertaken using PerkinElmer Frontier.

Tensile tests were conducted at room temperature using an INSTRON (Norwood, MA, USA) 3367 tensile machine with a 500 N load sensor. Testing speed was 10 mm/min. Each material, pristine and healed ones, is tested with at least 6 samples. Tensile samples were obtained from films using calibrated ISO 527-2 2B cutter. Samples used for the quantification of self-healing were manually cut in half and put in contact before healing thermal treatment, consisting in heating pristine and cut samples in an oven 2 h at 75 °C before tensile testing. A reprocessing test was conducted using a 50 t thermo-hydraulic press (PEI, Chalon-sur-Saône, France). Xerogel film was cut manually in submillimeter pieces that were pressed with 1 ton compression force for 4 h at 75 °C. Tensile test samples were then cut from the obtained film. Indentation marks were obtained using a HM-200 micro-Vickers hardness testing machine (MITUTOYO, Kanagawa, Japan). 9 indents were made on each coating by the application of respectively 0.01, 0.02, 0.03, 0.05, 0.1, 0.2, 0.3, 0.5 and 1 N. Scratch tests were performed using a Nano Scratch Tester CSM with a diamond indenter with conical geometry. We obtained 1 mm long scratches with the application of 100 mN loading force at 1 mm/min. Stress relaxation experiments were conducted using an Physica MCR 301 rheometer (Anton Paar, Graz, Austria) in shear mode with parallel plate geometries. We applied 5% strain on 2 mm diameter- and 1 mm-thick disks specimens and the relaxation modulus was monitored as a function of time.

EIS experiments were conducted in a three-electrode electrochemical cell connected to a SI 1286 Electrochemical Interface potentiostat and a SI 1260 Impedance/Gain-Phase-analyzer (Solartron Instrument, Bognor Regis, UK). The three-electrode cell was composed of a standard saturated calomel electrode, graphite as a counter electrode and coated AA2024-T3 aluminium plates as the working electrode. The surface of the coated aluminium plates was limited to 7 cm^2^ by applying a Teflon tape. Samples were immersed in a 0.05 M NaCl solution throughout the experiment. This concentration was used to obtain slow corrosion rates, as already reported in other studies on coatings exhibiting the same behavior [26,27]. All measurements were performed after stabilization (30 min) of open circuit potential (OCP). EIS spectra were registered from 10^−2^ to 10^5^ Hz with a 50 mV sinusoidal perturbation signal. Two samples for each formulation were evaluated simultaneously to ensure reproducibility of the results.

Thermogravimetric analyses (TGA) were carried out with a TG-DTA 92 equipment (Setaram, Fieux, France). Acquisition was performed under N_2_ from 25 to 700 °C, by heating steps of 10 °C/min. DSC measurements were performed under dynamic conditions under N_2_ gas flow, using a DSC 204 Phoenix Series apparatus coupled with a TASC 414/4 controller (Netzsch, Selb, Germany). Indium was used to verify the calibration. Analyzed samples were weighted with the accuracy of ±0.1 mg in aluminium capsules. Aluminium pans containing 15–20 mg of polymer were heated two times from −70 °C to 100 °C at a heating rate of 10 °C/min under a continuous flow of nitrogen. The glass transition temperature was determined during the second run. Proteus^®^ Software (Netzsch, Selb, Germany) was used to determine temperatures associated with glass transition, using Onset calculated temperature. Surface characterization was undertaken with an Interferometric Microscope Sensofar S-Neox (Sensofar, Barcelona, Spain) in white light mode and optical microscope 3D (Keyence, Osaka, Japan). TEM 70 nm slices were obtained by cutting at −80 °C using a EM FC7 Cryo-Ultramicrotome (Leica, Wetzlar, Germany). Samples were observed using a HT7700 (Hitachi, Chiyoda, Japan) at 80 kV on 100 formvar Carbon meshes.

## 3. Results and Discussion

### 3.1. Chemical and Thermal Characterizations of the Materials

**Figure 3:**
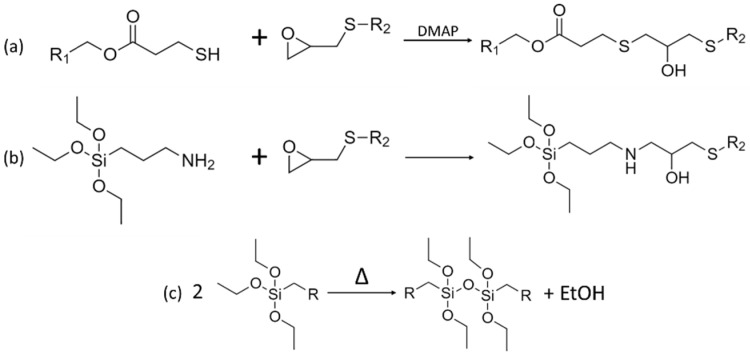
Crosslinking reactions taking place during synthesis: (**a**) thiol-epoxy, (**b**) epoxy-amine and (**c**) sol-gel condensation.

An organic-inorganic hybrid polysulfide material was synthesized using three different crosslinking chemical reactions, illustrated in Figure 3. The organic network was formed using thiol-epoxy and epoxy-amine reactions whereas the inorganic network was based on sol-gel condensation reactions. APTES, denoted as the hybrid crosslinker in this study, was used to covalently connect both organic and inorganic networks thanks to its silicon-carbon bond. Spectroscopic analysis methods were undertaken to identify the chemical structure of the materials, in order to verify that all precursor reacted after synthesis and to confirm the assumptions made during formulations design.

**Figure 4:**
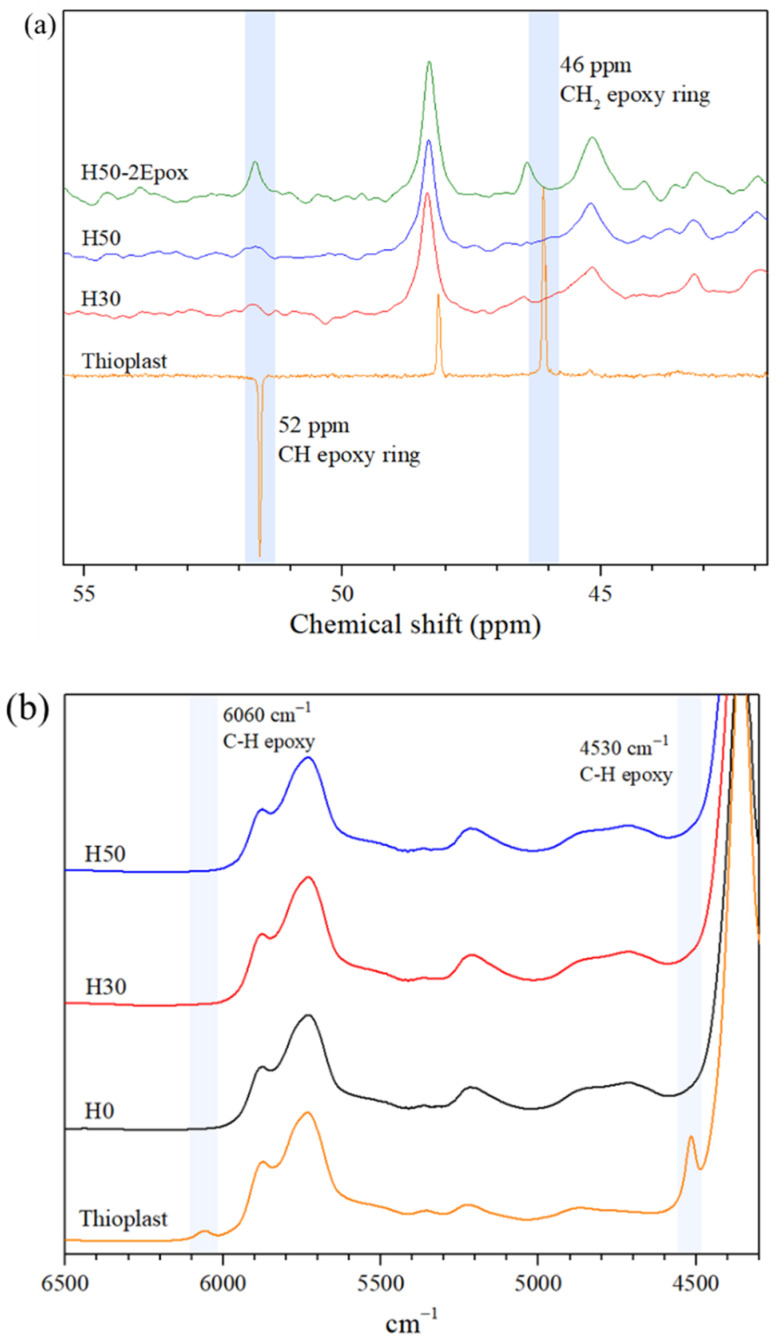
(**a**) ^13^C{^1^H} nuclear magnetic resonance (NMR) in acetone-D6, ^13^C CPMAS and (**b**) nIR spectrum of pure Thioplast, H0, H30 and H50 films.

NMR studies were conducted on pure Thioplast, H50 and H50-2Epox to detect epoxide rings reactions. As explained earlier in Section 2.1, those experiments were conducted to observe the reactivity of secondary amines of APTES. Thioplast EPS-25 being liquid and H50 and H50-2Epox being solid, ^13^C{^1^H} NMR inacetone-D6 and ^13^C CP-MAS NMR were respectively used for their characterization and are compared in Figure 4a. Two peaks at 46 and 52 ppm present for pure Thioplast, were assigned to CH_2_ and CH carbon atoms of the epoxide ring respectively. These peaks were not visible on standard formulations H30 and H50 but were still present in the spectrum of the H50-2Epox sample. This indicated that secondary amines of APTES of H50-2Epox materials might not have fully reacted with available epoxide rings. This result was in agreement with the macroscopic observations of the films, H50-2epox being much stickier and softer than H50. If secondary amines reacted completely a denser and more reticulated polymeric network would have been obtained. Based on that result, all materials presented in this study, except H50-2Epox, were synthesized by considering only the reaction of primary amines in their formulation.

The reaction of epoxide groups of Thioplast-EPS25 was monitored using near infrared spectroscopy to confirm their total reaction in H0, H30 and H50 “standard” formulations. In the nIR spectra shown in Figure 4b, characteristic stretching vibrations the oxirane ring at 4530 and 6080 cm^−1^ respectively can be observed in the pure Thioplast spectrum [28,29]. As already observed with ^13^C CPMAS NMR measurements, none of them are present for H0, H30 and H50 films. These results indicate that all epoxide functions reacted with 4SH or APTES crosslinkers, giving rise to the organic polymeric network.

**Figure 5:**
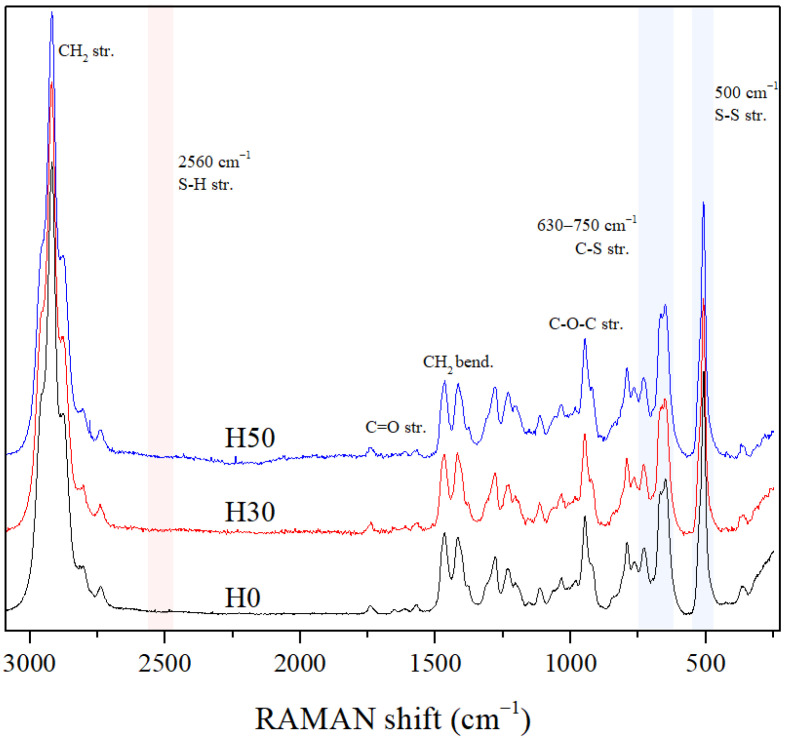
RAMAN spectra of H0, H30 and H50.

RAMAN spectra of organic and hybrid films H0, H30 and H50 are visible in Figure 5. All spectra are very similar. This spectroscopy allows unambiguous identification of a thiol functional group by the presence of the very intense SH stretching vibration at 2560 cm^−1^. The complete reaction between the organic crosslinker 4SH and the Thioplast monomer is confirmed since this peak is not present in any spectra of all studied samples. Sulfur related vibrations at 500 (S-S bending mode), 630 and 700 cm^−1^ (C-S stretching modes) [30] appeared in all spectra, confirming the presence of disulfide bonds of Thioplast in the polymeric network.

**Figure 6:**
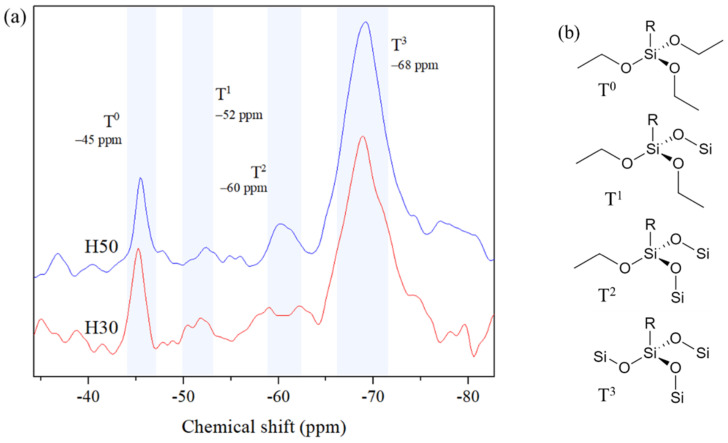
(**a**) ^29^Si magic angle spinning (MAS) NMR spectrum of H30 and H50 hybrid films and (**b**) representation of siloxane condensation degrees.

^29^Si CP MAS-NMR was used to monitor the formation of the inorganic network by characterizing the condensation degree of APTES in hybrid films. Figure 6a present spectra of H30 and H50, both exhibiting peaks at −52, −60 and −62 ppm corresponding to siloxane bonds with different condensation degrees, schematically represented on Figure 6b. As in previous spectroscopic measurements, their spectra are very similar. The peak with the highest area, more than 85% of total peak area, is the one at −62 ppm corresponding to fully condensed silicon atom type (T^3^). Only few T^1^ and T^2^ silicon atom types are visible in both spectra. Some non-hydrolyzed trialkoxysilane T^0^ are also visible, indicating that the inorganic network is not completely condensed, which might be explained by the absence of water or any catalyst for sol-gel reactions of APTES in the formulation. Silane condensation might be favored by the thermal treatment giving rise to the formation of the inorganic siloxane network, the presence of alkoxyde functions in the polymeric backbone or by the residual water present in the solvent. T^0^ occurrence can also be explained by the presence of APTES at the extremity of some polymer chains, trapped in the organic matrix and unable to react with other alkoxysilanes. Silicon with low condensation degree can also react with alcohol functions obtained after epoxy ring opening and form new Si–O–C bonds. All those results confirm the production of hybrid polymeric materials mixing organic and siloxane networks.

Thermal properties of films were studied using ATG and DSC measurements and are reported in Table 2; the corresponding curves are presented in Supplementary Materials, Figure S1. More than 75% of the materials decomposes at 275 °C, attributed to the decomposition of the organic network. This decomposition continues with increasing temperature, with less than 5% left in all samples at 650 °C. A difference is observable between organic and hybrid samples after 300 °C, as hybrids films seems to decompose slower. This behavior difference can be linked to the presence of more thermally stable Si–O–C and Si–O–Si bonds. It is considered that only siloxane bonds of the inorganic network are still present at 650 °C, so residual weight at this temperature can be seen as a measurement of weight percentage of inorganic network in the material. As shown in Table 2, measured weights are in good agreement with theoretical siloxane values calculated from formulation amounts. DSC measurements reveal quite the same behavior, all three polymers having almost the same glass transition temperature with respect to the precision of the measurement. It is also important to notice that only the glass transition is visible and no reaction peak was detected, in agreement with the spectroscopic data attesting that the materials are fully reacted.

### 3.2. Self-Healing of Films and Coatings

**Figure 7:**
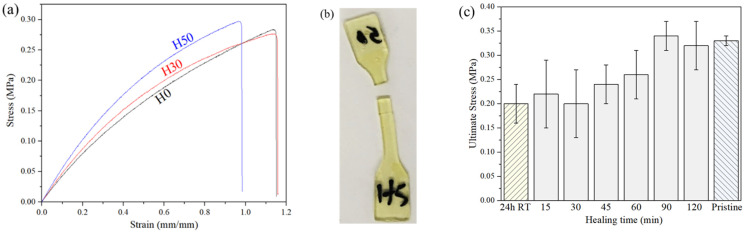
(**a**) Stress-Strain curves of pristine H0, H30 and H50 samples, (**b**) photograph of a H50 tensile sample where fracture occurred at a different place than original wound, (**c**) measured stress at break for wounded H50 samples after different healing times at 75 °C or 24 h at room temperature.

Combining mechanical strength and self-healing ability is one of the main challenges for this kind of adaptive covalent network polymers. Adding strong and tridimensional inorganic bonds in the material network could be one solution to obtain such materials. To evaluate the effect of the introduction of APTES on both mechanical and self-healing properties of the materials, tensile stress-strain measurements were conducted on H0, H30 and H50 dogbone ISO 527-2-5B samples. Some samples were cut in halves, brought in contact manually, and underwent healing thermal treatment for 2 h at 75 °C. In order to ensure comparable results, unwounded samples undergo a similar heating of 2 h at 75 °C because of the possible modifications of the inorganic network during that thermal treatment. Tensile tests results for pristine and healed samples are presented on Table 3, showing Young’s modulus, breaking strain and stress at break for H0, H30 and H50 samples. As also visible in Figure 7a presenting the stress-strain curves of the materials, it appears that the introduction of APTES seems to make materials stiffer, slightly increasing their elastic modulus and reducing their breaking strain. Our results are in accordance with other measurements on the same organic polysulfide published earlier [9,14].

The slight stiffening of the materials do not seem to have a detrimental effect on self-healing properties as a full recovery of breaking strain and stress at break is obtained after 2 h at 75 °C for all formulations with respect to the accuracy of the measurement. Young’s modulus of every tested sample is really close to pristine values, attesting to the good reconstruction of the polymeric network between previously separated parts. Samples breaking at a different position than the original wound, as illustrated in Figure 7b, are observed in every formulation proving the resistance of the healed interface. However, it is important to mention the importance of initial placement of the two halves during self-healing measurement. A little misplacement at the interface can lead to incomplete healing and early break of the sample during the test, as already mentioned by Pepels et al. [14] who showed that weaker samples are obtained when the two surfaces are not perfectly attached. This fact combined with other factors like the relative smallness of samples or the sample preparation by cutting could explain the quite high relative errors in tensile results.

Tensile tests were also conducted on wounded H50 samples with different healing time and temperature. As shown in Figure 7c, full recovery is achieved after 90 min at 75 °C. It is interesting to note that for lower healing times a significant adhesion between parts and tensile strength is obtained, but not as high as pristine samples. After 24 h at room temperature the same noticeable adhesion is present and early breaking is observed. As noticed in similar disulfide-based self-healings polymers, temperature seems to have a significant effect on the recovery process. This effect can be explained by the higher polymer chains mobility and the disulfide metathesis reactions enabled by the rise of the temperature.

**Figure 8:**
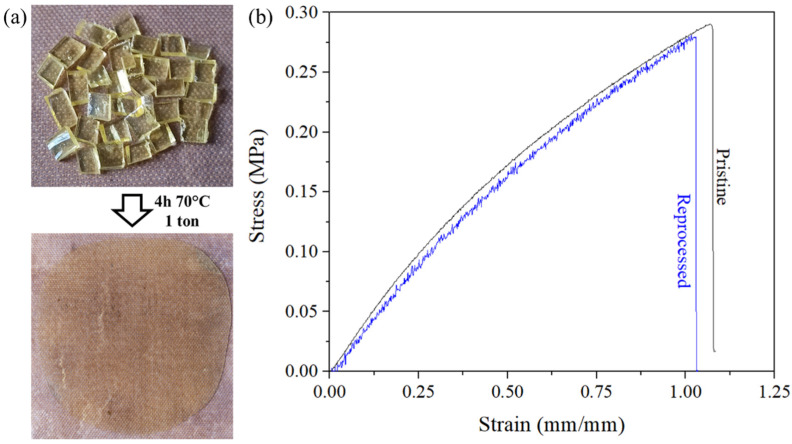
(**a**) Digital images of H50 film before and after reprocessing by hot pressing 4 h at 75 °C, (**b**) stress-strain results of pristine and reprocessed H50 samples.

Reprocessing tests were conducted by pressing fragments of H50 films at 1 ton during 4 h at 75 °C. A thin, transparent and homogenous film was obtained as shown in Figure 8. While being much thinner than pristine tested samples of previous tests (0.3 mm against 2 mm for usual samples), reprocessed samples exhibit almost the same tensile properties with respect to the precision of the measurements. The small loss of maximum strain could be attributed to the permanent loss of non-recoverable bonds in the material.

In order to observe the self-healing behavior of coatings, two different wound morphologies were studied. Mechanical testing machines were used to obtain repeatable scars whatever the sample or the formulation used. Holes were made by the application of increasing indent forces from 0.01 N to 1 N using a microindentor mechanical testing machine to obtain increasing wound dimensions. Scratches were made using a nanoscratch mechanical machine by the application of 100 mN pressure. Unfortunately, due to the low hardness of the samples obtained, the scratches are not as clean and identical as desired, but it still gives a very good indication of the self-healing abilities of the coatings.

**Figure 9:**
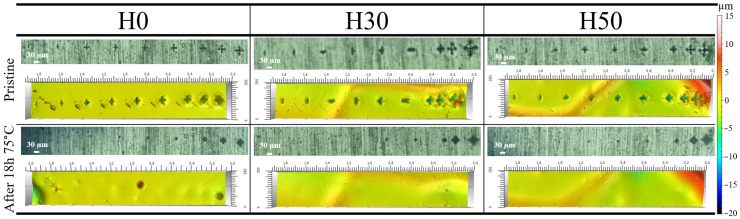
Optical and interferometric surface analysis results of indented coatings before and after 18 h at 75 °C.

Figure 9 shows surface morphology of tested coatings by optical microscopy and interferometric surface measurements before and after 18 h at 75 °C. Before thermal treatment all three coatings exhibit indenter marks of progressively growing dimensions due to increasing testing force. After heating, none of those marks are still visible on the surface of the coatings, while the marks on the aluminium substrate are still visible through it thanks to the transparency of the coating material. Even the largest wounds, 2 times larger than the coating thickness, were healed after the healing thermal treatment. It is important to mention that the healing time of 18 h was arbitrarily chosen for practical reasons, so it is possible that full healing might be obtained with shorter times.

**Figure 10:**
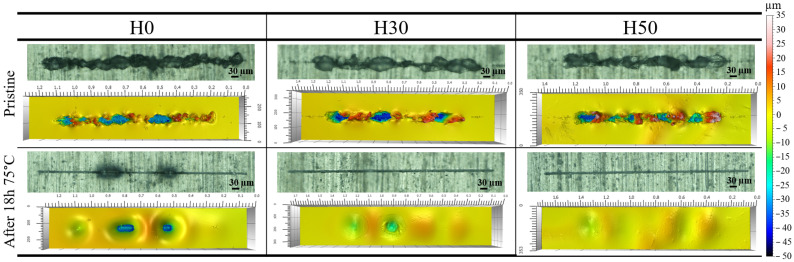
Optical and interferometric surface analysis results of scratched coatings before and after 18 h at 75 °C.

Figure 10 shows scratched coating surfaces morphology before and after healing treatment of 18 h at 75 °C. After healing scratches on hybrid H30 and H50 coatings are fully closed, while the substrate is still exposed at some parts of H0 coating. Like for indenter marks, scratch imprints on the substrate are still visible through coatings but interferometric surface studies confirm the recovery of their surface relative flatness. Those tests confirm previous statements but comparison between samples is more difficult because of the differences between initial scratches morphology. Hybrid materials seems to have faster healing properties, but this observation needs to be considered carefully because of the original scratch diensions’ differences. As the profile extracted from surface data shown in red in Figure S2 exhibits, H50 coatings are capable of healing wounds as wide as twice its thickness.

### 3.3. Anticorrosive Properties of Coatings

**Figure 11:**
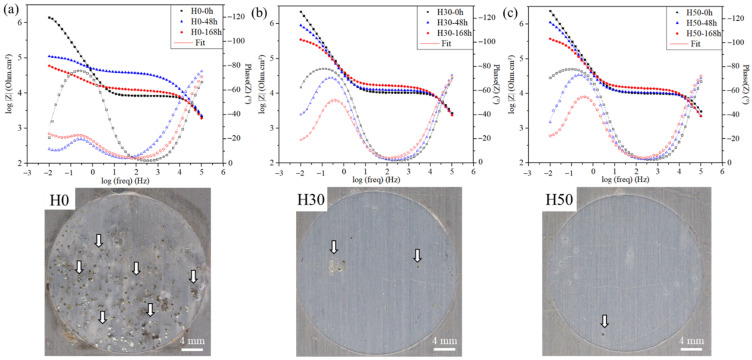
Electrochemical impedance spectroscopy (EIS) spectra after different immersion times in 0.05 M NaCl solution and photograph of the surface of samples after one week of immersion of (**a**) H0, (**b**) H30 and (**c**) H50 coatings, arrows show examples of corrosion pits.

The protection against corrosion of these coatings were investigated using EIS after their immersion in 0.05 M NaCl solution. This concentration was chosen in order to discriminate the behaviors of the different coatings. Bode plots of H0, H30 and H50 at different immersion times are presented in Figure 11a–c respectively, with impedance points represented in filled symbols and phase angle in hollow ones. Analyses were conducted every 24 h of immersion, from 0 h to 168 h. The three coatings show high impedance values above 10^6^ Ω.cm^2^ at low frequencies at 0 h immersion. Two time constants can be seen in the phase angle plot, one at 5.10^2^ Hz and another around 0.5 Hz. They can be ascribed, respectively, to the responses of the coating and of the oxide layer at the interface coating/substrate. As reported in the literature [31,32] those spectra profiles indicate the relative permeability of the coatings to the electrolyte, probably because of its chemical composition. Moreover, in the case of H0, a low-frequency (0.05 Hz) time constant appears due to the start of corrosion activity on the surface of the substrate. Its surface shows accordingly many corrosion products after 1 week of immersion as shown in Figure 11 First corrosion products were visible after only 48 h of immersion, in accordance with the EIS spectrum. This third time constant is not visible for both H30 and H50 hybrid coatings except maybe after 168 h of immersion, as their surface almost free from corrosion products seems to corroborate. The presence of the corrosion pits according the immersion time is highlighted on the photographs of the exposed surface (3 cm diameter) in Figure 11 with white arrows.

**Figure 12:**
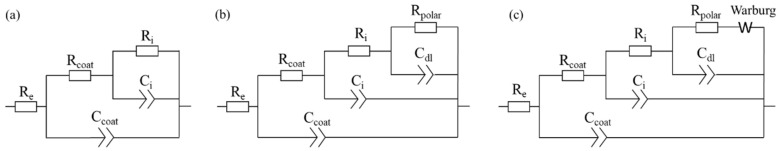
Equivalent circuit models (**a**–**c**) used for EIS data fitting.

Equivalent circuit modeling was used to investigate more deeply the differences between coatings and to monitor the evolution of their properties. Constant phase elements (CPE) were used instead of capacitors in all fittings presented in the study to account for the inhomogeneity of dielectric layers. The equivalent circuits chosen for fittings were based on the number of time constants and in order to maximize the goodness of fits. Equivalent circuits presented in Figure 12, already used in numerous studies for hybrid protective coatings [31,32,33], were used for numerical fitting of the experimental Bode plots. Circuit (a) was used for short immersion times, when only two electrochemical responses were observed. Coating’s resistance (R_coat_) and CPE (Q_coat_) were assigned to the first time constant observed at high frequencies. Resistance (R_i_) and dielectric properties (Q_i_) of the interface layer were assigned to the response at low frequencies. For longer immersion times and when the electrolyte reaches through the substrate, a third time constant is present and circuit (b) assessing the polarization resistance (R_polar_), double layer CPE (Q_dl_) was used. A Warburg element was added for circuit (c) to account for the corrosion process at the longest immersion times of H0. Fit results are represented on the Bode plots presented in Figure 11.

Coating resistance and CPE values obtained with equivalent circuit modelling are presented in Figure S3. Hybrid coatings exhibit a slightly higher resistance than H0, but this difference is quite small. Interestingly, R_coat_ values slightly increase with time for all samples. Meanwhile, their CPE values increases with immersion time because of the accumulation of charged species on the coating surface. The incorporation of APTES in the formulation seems to attenuate this effect slightly.

**Figure 13:**
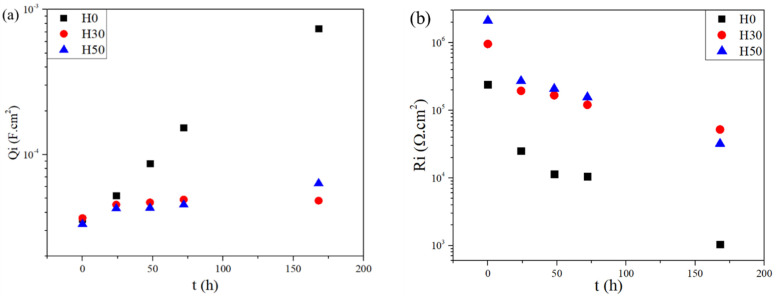
Evolution of (**a**) constant phase elements (CPE) value of the interface Q_i_ and (**b**) its resistance R_i_ during immersion in 0.05 M NaCl, calculated using equivalent circuits modelling.

Anyway, a clear difference is observable with the behavior of the coating/substrate interface. Figure 13 presents the evolution of R_i_ and C_i_ with immersion time. Pure organic coating H0 CPE value increases by more than a decade after one week of immersion while H30 and H50 hybrid coatings show a much smaller increase. This sharp increase for the organic coating seems to indicate its quick delamination with immersion time. The lower increase for hybrid coatings could be explained by the presence of hybrid crosslinkers in the formulations, able to form a dense interface layer between the coating and the substrate. Indeed, the ability of the siloxane part of APTES to form strong covalent Si–O–Al bonds with the surface to obtain resistant and adhesive protective layers have already been reported in studies on similar hybrid coatings [31,34,35]. This enhancement of the protective properties at the interface is highlighted by their resistance values R_i_. While a similar decrease with immersion time is seen in all tested coatings, hybrid layer resistance values are higher by more than one decade at 0 h and two decades after 168 h. It is this difference of behavior at the interface, thanks to the best chemical compatibility coating/substrate, that is thought to enhance of the anticorrosive properties of the coating when hybrid crosslinker is present.

**Figure 14:**
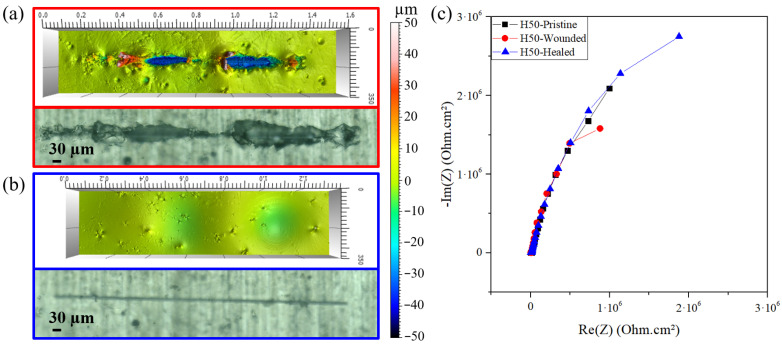
Optical and interferometric analysis of (**a**) scratched and **(b**) healed H50 coating after 18 h at 75 °C, (**c**) Nyquist plots of pristine, scratched and healed H50 coating.

While being more protective than pure organic coatings, H50 hybrid coating also possesses self-healing properties, as shown earlier in part 3.2. A H50 coating was scratched and healed, with EIS analysis at each step to observe the effect of the healing process on the electrochemical properties of the layer. Figure 14a,b shows the surface aspect of tested coating before and after healing treatment of 18 h at 75 °C, demonstrating the complete closure of the original scratch. As visible in Figure 14c Nyquist plots, healing led to the complete restoration of the electrochemical properties of the protective layer. An enhancement of the protective properties even seems to take place, probably because of the post condensation of some of the unreacted alkoxysilane moieties due to the long thermal treatment. This result highlights the potential of intrinsic self-healing layers to recover their protection properties after damage to obtain performant coatings against corrosion.

### 3.4. Discussion on the Effect of the Hybrid Crosslinker

**Figure 15:**
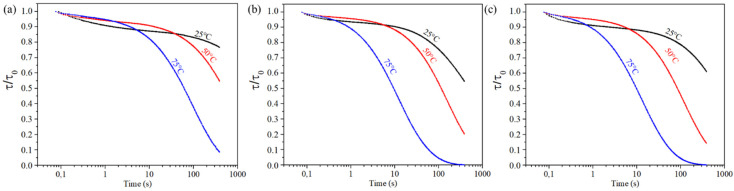
Stress relaxation of (**a**) H0, (**b**) H30 and (**c**) H50 films at 25 °C (black), 50 °C (red) and 75 °C (blue).

Stress relaxation experiments were realized in order to better understand the consequences of the addition of the hybrid crosslinker on films properties. By measuring the evolution of applied stresses over time, those experiments provided good information on the intrinsic mobility of the polymeric networks. Because the materials are able to relax stresses thanks to the presence of the disulfide bonds [17], those measurements were also a good indicator of the self-healing potential of the films. The faster the relaxation occurs, the faster the healing process will be. Stress relaxation plots of H0, H30 and H50 at 25, 50 and 75 °C are presented in Figure 15.

H0 films exhibit the capability to relax stress with time, as depicted in other studies based on the same polymeric material [17]. Interestingly H30 and H50 hybrid films are also capable of relaxing applied stresses, and even faster than the H0 organic film. The higher the temperature, the faster the process will be. All tested films are capable to fully or almost fully relax applied stress within 300 s at 75 °C. This result confirms the use of this temperature for our previous self-healing studies. The inorganic network formed by sol-gel reactions seems then not to hinder the intrinsic molecular mobility of the chains. This result differs from the work described in the bibliography [36,37,38] by the low level of siloxane bonds introduced, which in our case reaches a maximum of 2.8% by mass. Another distinction also comes from the microstructure of the material and the distribution of the different polymer networks obtained with the in situ one-pot synthesis implemented in our work.

**Figure 16:**
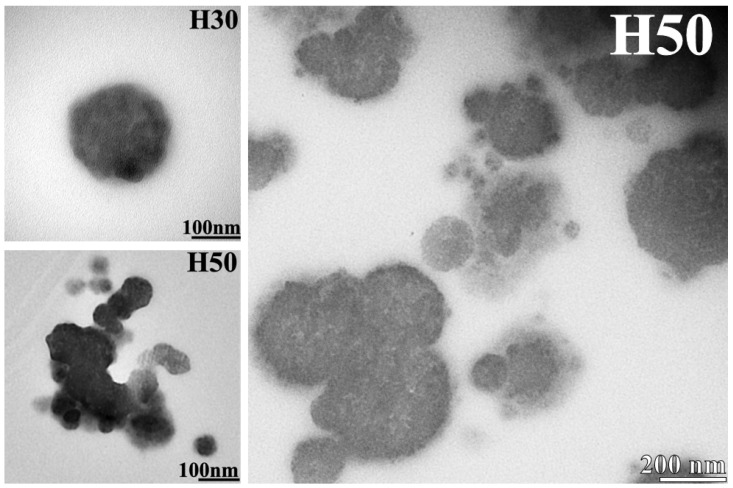
H30 and H50 transmission electron microscopy (TEM) micrographs at different magnifications.

Transmission electron microscopy (TEM) observations were undertaken on 70 nm hybrid film slices H30 and H50 to observe the repartition of the inorganic network in the materials. Figure 16 shows representative micrographs obtained for H30 and H50 films. Spherical dark domains of aggregated spheres with different diameters are visible on both films, corresponding to the inorganic siloxane domains in the materials. In contrast, the organic matrix can be seen as the lighter phase around those dark domains. None of those dark spheres are visible in H0 micrographs, which confirm their attribution as inorganic domains formed by APTES condensation.

APTES seems, then, to be present mostly in aggregates dispersed randomly in the organic network, forming a nanocomposite-like microstructure. This microstructure was already observed in previous studies conducted by Matějka et al. [39,40] on similar epoxy-silica hybrid materials, synthesized using the same simultaneous one step polymerization method of the inorganic and organic polymeric networks. They attributed this large nanodomain formation to the high presence of amine moieties in the formulation implying nucleophilic catalysis of the sol-gel reactions and fast polycondensation reactions compared to hydrolysis. This assumption could also be made in our case thanks to the presence of the amine functions of APTES. Phase separation occurring during synthesis could also contribute to those spherical particles’ formation. This large nanodomains formation could be an explanation why the incorporation of APTES has no or little effect on the glass transition temperature. Studies have indeed showed that the modification of the properties of organic polymers is the lowest for nanocomposites when the inorganic domains are the largest, due to the reduction of the interfacial area between phases [41,42].

**Figure 17:**
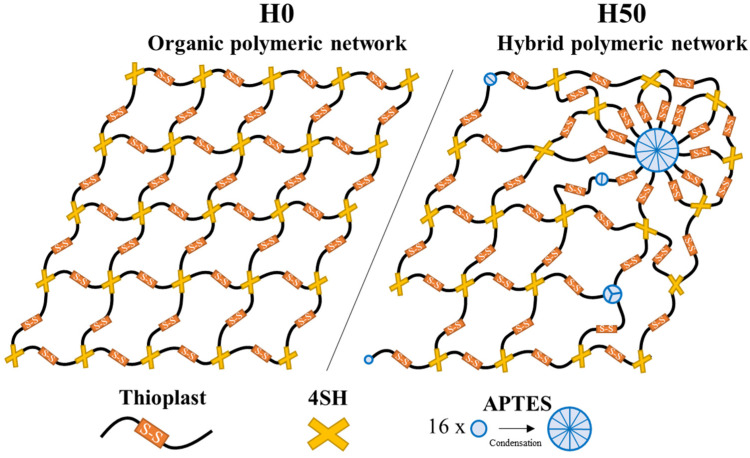
Schematic representation of polymer networks for H0 and H50 materials.

H30 and H50 hybrid films are then composed of spherical inorganic nanodomains dispersed in an organic self-healing matrix. Song et al. [17] proved that the presence of “non-healable” domains in a polydisulfide matrix is not detrimental to the self-healing properties, even with only a low molar fraction of the monomers containing disulfide bonds. The formation of those large inorganic domains in the materials can, therefore, be an explanation why H30 and H50 films still possess self-healing properties. By replacing 4SH in the network while being much more aggregated, the insertion of APTES might greatly modify the homogeneity of the polymeric structure. Formation of these nanodomains bearing a concentration of amino functional group at their surface might help disulfide bonds to be closer to each other, facilitating their metathesis reaction. The attempt of a graphical representation of organic H0 and hybrid H50 networks is proposed in Figure 17. While the H0 polymeric network could be quite homogeneous, the presence of the inorganic crosslinkers might disrupt this harmony, modifying crosslink density. As APTES condenses and forms relatively large particles, new crosslinking points with higher functionality than with 4SH are created. On the other hand, as shown with NMR measurements, some T^0^ uncondensed silane are still present, leading to end points in the polymeric network and more space between chains. It is this disruption of the polymeric network that is believed to contribute to the self-healing properties of tested hybrid materials in spite of the presence of the inorganic nanodomains.

## 4. Conclusions

The replacement of the organic crosslinker 4SH by APTES as a hybrid organic-inorganic crosslinker was studied in a disulfide-based self-healing polymer, focusing on the anticorrosion and self-healing properties of coatings and films. The obtained hybrid films were slightly more rigid, with a higher Young’s modulus but no influence on thermal properties was observed. All obtained materials were able to recover their tensile properties upon heating for 2 h at 75 °C and the presence of the hybrid crosslinker had no detrimental effect on this essential property. Hybrid films could even be reprocessed by heat pressing and exhibit the same properties as pristine samples. Spherical inorganic nanodomains embedded in an organic self-healing matrix were obtained in the films, forming a nanocomposite-like structure. This particular structure and the modification of the crosslinking density of the polymeric network led to a slightly faster capability of hybrid films to relax stress compared to the organic polymer, probably due to a high concentration of disulfide bonds next to the nanoparticles facilitating the metathesis reactions.

The presence of the hybrid organic-inorganic crosslinker in the coatings improved the anticorrosion properties thanks to the formation of a hybrid layer between the coating and the AA2024-T3 substrate. The slight addition of APTES allowed us to obtain coatings showing little or no corrosion products after 1 week of immersion in NaCl corrosive solution, while the pure organic coating was completely corroded. Both organic and hybrid coatings were able to restore their original surface aspect after healing at 75 °C for different wounds morphologies. Hybrid samples were able to heal scratches more than two times wider than the coating’s thickness. This wound closure permitted the full recovery of the protective properties of the coatings.

Employed here with an organic coating with low barrier properties, the slight introduction of hybrid organic-inorganic crosslinker in self-healing organic polymers was proven to be a promising way to enhance the properties of organic protective coatings. Given the wide choice of precursors, this methodology can easily be transferred to other chemical materials to produce high-performance anticorrosion and self-healing coatings.

## Figures and Tables

**Table 1:** Crosslinker molar ratios of the four tested materials.

	$\frac{4\mathrm{SH}}{\mathrm{Thioplast}}(\%\;\mathrm{mol})$	$\frac{\mathrm{APTES}}{\mathrm{Thioplast}}(\%\;\mathrm{mol})$	$\frac{\mathrm{APTES}}{\mathrm{APTES}+4\mathrm{SH}}(\%\;\mathrm{mol})$	DMAP (% Total Weight)	EtOAc (% vol)
H0	0.50	0.00	0.00	0.1	0.42
H30	0.46	0.21	0.30	0.1	0.42
H50	0.42	0.42	0.50	0.1	0.42
H50-2Epox	0.34	0.35	0.50	0.1	0.42

**Table 2:** Glass transition temperatures and weight loss for H0, H30 and H50 films.

Material	T_g_ (°C)	10% Weight Loss (wt%)	w% at 650 °C (%)	Theoretical SiO_2_ Values (wt%)
H0	−51 ± 2	255	0.2	0
H30	−53 ± 2	250	1.4	1.3
H50	−50 ± 2	248	2.3	2.8

**Table 3:** Tensile tests result for H0, H30 and H50 after 2 h at 75 °C, without and with prior complete separation.

Material	Young’s Modulus (MPa)		Breaking Strain (%)		Stress at Break (MPa)	
	Pristine	Healed	Pristine	Healed	Pristine	Healed
H0	0.42 ± 0.03	0.43 ± 0.03	112 ± 9	100 ± 25	0.29 ± 0.02	0.27 ± 0.04
H30	0.46 ± 0.03	0.48 ± 0.02	113 ± 12	83 ± 17	0.29 ± 0.03	0.25 ± 0.03
H50	0.53 ± 0.06	0.53 ± 0.05	105 ± 15	100 ± 20	0.32 ± 0.05	0.31 ± 0.06

## Data Availability

The data presented in this study are available on request from the corresponding author.

## Supplementary Materials

The following are available online at https://www.mdpi.com/article/10.3390/ma14185382/s1. Figure S1. (a) ATG and (b) DSC of H0, H30 and H50 films, Figure S2. Longitudinal scratches profiles extracted from interferometric surface analysis of H0 and H50 coatings before and after 18 h at 75 °C healing treatment. Figure S3. Evolution of (a) Ccoat and (b) Rcoat of the coatings during immersion in 0.5 M NaCl.
