# Supplementary material for: Influence of Hybrid Sol-Gel Crosslinker on Self-Healing Properties for Multifunctional Coatings
Source: Materials (Basel). 2021 Sep 17;14(18):5382. doi: 10.3390/ma14185382 (PMC8465503; doi:10.3390/ma14185382)
Supplement: Supplementary file 1 [file materials-14-05382-s001.zip › materials-1379203-supplementary.pdf]

Influence of Hybrid Sol-Gel Crosslinker on Self-Healing Properties for Multifunctional Coatings

Guillaume Lollivier [1], Marie Gressier [1], Florence Ansart [1], Maëlenn Aufray [2] and Marie-Joëlle Menu [1,*]

Supplementary Materials

Figure S1. (a) ATG and (b) DSC of H0, H30 and H50 films

Figure S2. Longitudinal scratches profiles extracted from interferometric surface analysis of H0 and H50 coatings before and after 18 h at 75°C healing treatment

Figure S3. Evolution of (a) C_{coat} and (b) R_{coat} of the coatings during immersion in 0.05 M NaCl

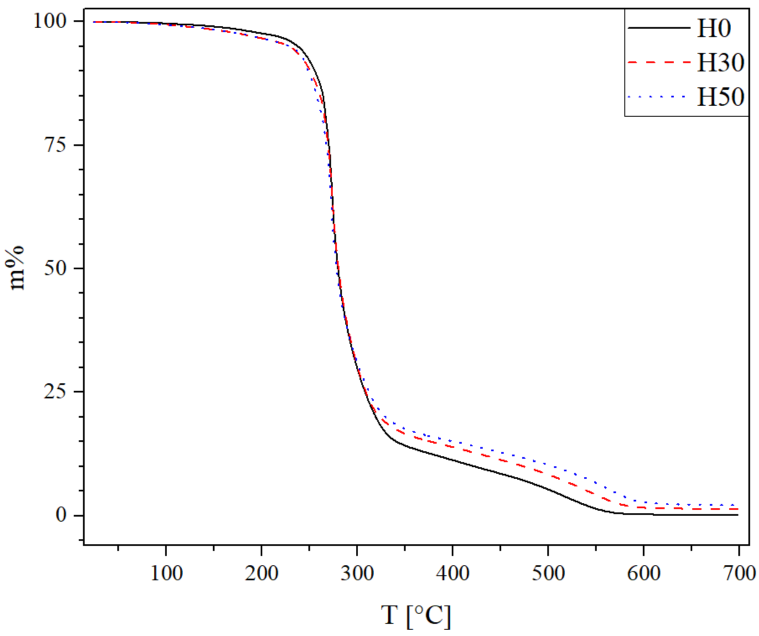

(a)

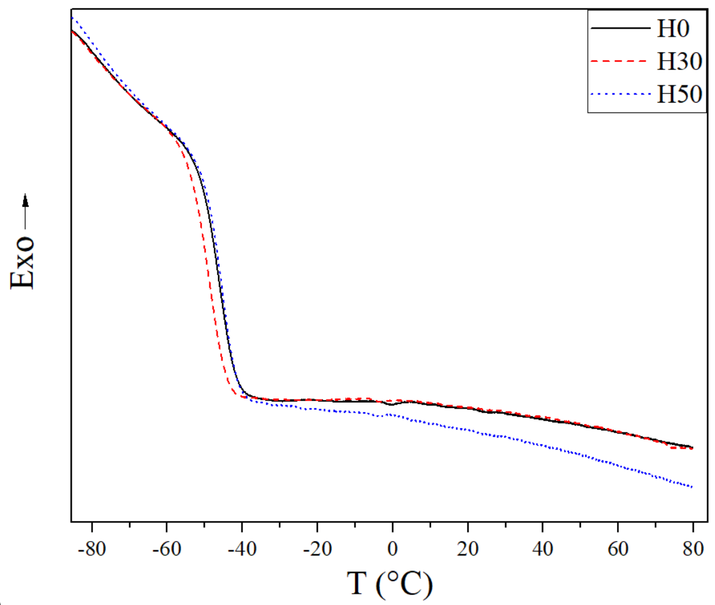

(b)

Figure S1. (a) ATG and (b) DSC of H0, H30 and H50 films.

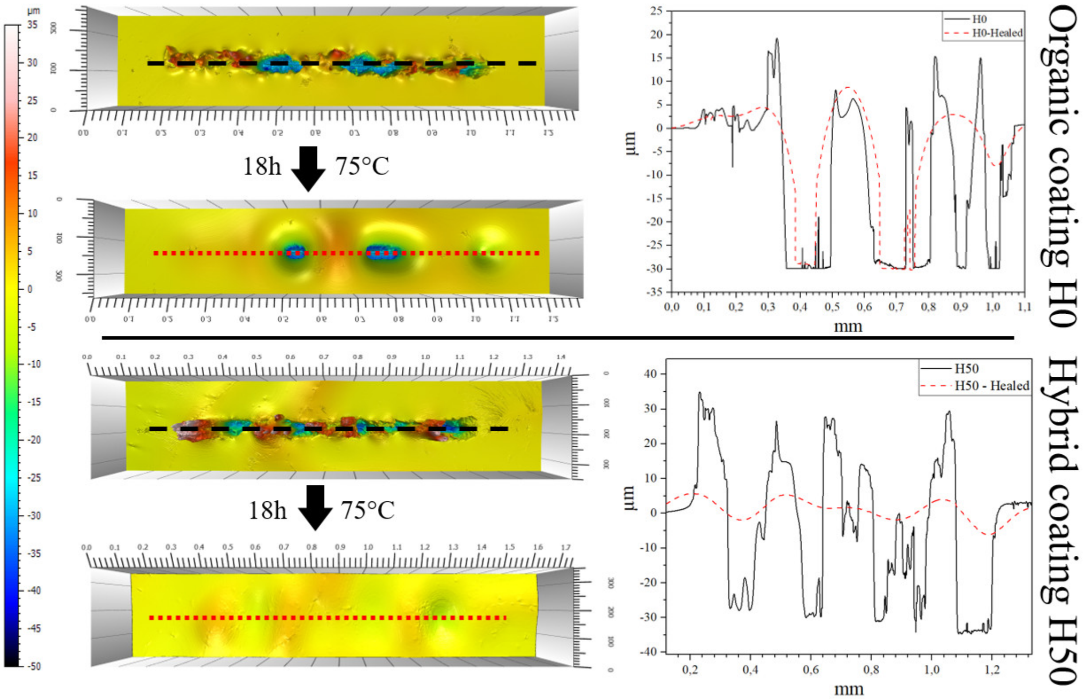

Figure S2. Longitudinal scratches profiles extracted from interferometric surface analysis of H0 and H50 coatings before and after 18 h at 75°C healing treatment.

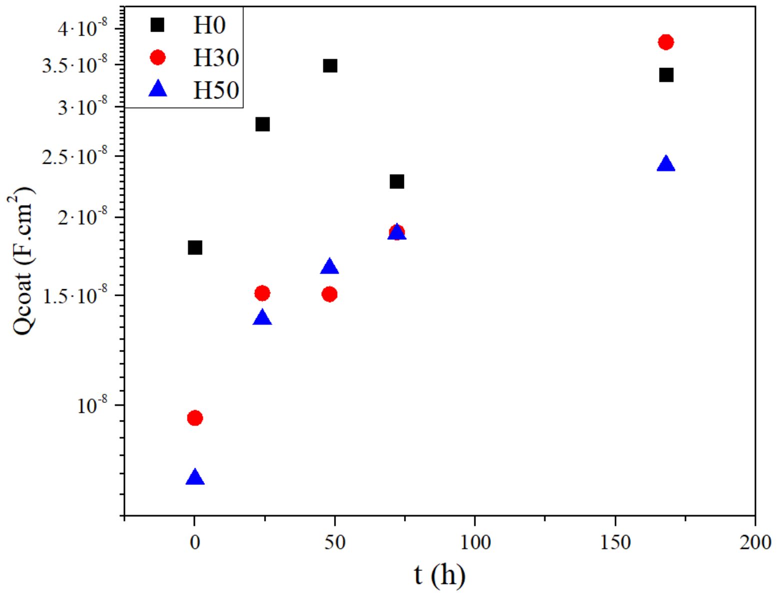

(a)

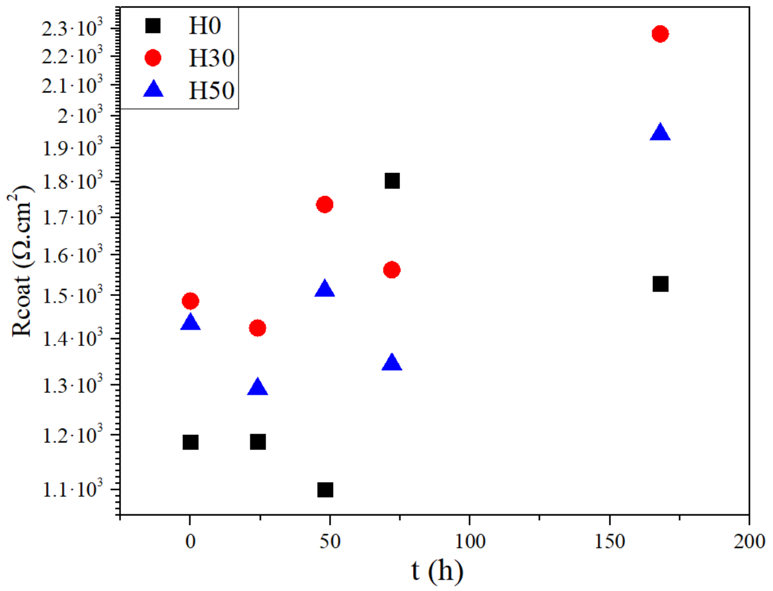

(b)

Figure S3. Evolution of (a) Qcoat and (b) Rcoat of the coatings during immersion in 0.05 M NaCl.
